# A Study on the Association Between Treatment Response to Atezolizumab Plus Bevacizumab Combination Therapy for Advanced Hepatocellular Carcinoma and Gut Microbiota

**DOI:** 10.3390/microorganisms14040867

**Published:** 2026-04-12

**Authors:** Yusuke Tanaka, Daiki Miki, C. Nelson Hayes, Michihiko Kawahara, Saki Sueda, Tomoaki Emori, Kou Hashimoto, Yuri Mitamura, Aiko Tanaka, Keiichi Hiraoka, Yusuke Johira, Ryoichi Miura, Hatsue Fujino, Atsushi Ono, Eisuke Murakami, Tomokazu Kawaoka, Masataka Tsuge, Shiro Oka

**Affiliations:** 1 Department of Gastroenterology, Graduate School of Biomedical and Health Sciences, Hiroshima University, Hiroshima 734-8553, Japan; y5186@hiroshima-u.ac.jp (Y.T.); nelsonhayes@hiroshima-u.ac.jp (C.N.H.); mitumitu@hiroshima-u.ac.jp (M.K.); suesaki@hiroshima-u.ac.jp (S.S.); t8emori@hiroshima-u.ac.jp (T.E.); k-hashimoto@hiroshima-u.ac.jp (K.H.); mitam@hiroshima-u.ac.jp (Y.M.); atanaka1@hiroshima-u.ac.jp (A.T.); khiraoka@hiroshima-u.ac.jp (K.H.); jyusuke9@hiroshima-u.ac.jp (Y.J.); ryoichim@hiroshima-u.ac.jp (R.M.); fujino920@hiroshima-u.ac.jp (H.F.); atsushi-o@hiroshima-u.ac.jp (A.O.); emusuke@hiroshima-u.ac.jp (E.M.); kawaokatomo@hiroshima-u.ac.jp (T.K.); tsuge@hiroshima-u.ac.jp (M.T.); oka4683@hiroshima-u.ac.jp (S.O.); 2 Liver Center, Hiroshima University Hospital, Hiroshima 734-8551, Japan

**Keywords:** hepatocellular carcinoma, gut microbiota: immune checkpoint inhibitor, atezolizumab plus bevacizumab combination therapy, treatment response, progression-free survival

## Abstract

Recent findings suggest that the gut microbiota modulates antitumor immunity and influences the efficacy of immune checkpoint inhibitors, yet no established consensus has been reached. This study examined the association between gut microbiota composition before initiation of atezolizumab plus bevacizumab combination therapy (Atez/Bev) and treatment response in patients with advanced hepatocellular carcinoma (HCC). Twenty-nine patients with advanced HCC from whom stool samples were collected before Atez/Bev treatment were enrolled. A comparative analysis was performed between the responder and non-responder groups to identify the genera associated with treatment response and progression-free survival (PFS). A total of 90 genera were identified. ROC analysis was performed for the responder and non-responder groups using the relative abundance of each genus. The prevalence of *Faecalibacterium* was significantly correlated with the responder group. Furthermore, multivariate analysis incorporating clinical prognostic factors also showed a statistically significant correlation between the prevalence of *Faecalibacterium* and the responder group. Analysis of the association with PFS revealed significantly prolonged PFS in the *Acidaminococcus*-high, *Megamonas*-high, *Lachnoclostridium*-low, and *Flavonifractor*-low groups. Multivariate analysis for PFS also confirmed significant correlations with the prevalence of *Acidaminococcus* and *Megamonas*. Our results suggest that gut microbiota may be associated with the efficacy of Atez/Bev treatment for advanced HCC.

## 1. Introduction

Hepatocellular carcinoma (HCC) is the most common primary liver malignancy, accounting for approximately 80–90% of all liver cancers. It ranks as the third leading cause of cancer-related deaths worldwide, claiming approximately 800,000 lives annually [1,2,3]. Immune checkpoint inhibitors (ICIs) have emerged as a novel therapeutic strategy for HCC by enhancing the antitumor immune response [4]. While favorable treatment outcomes have been achieved in some patients, the overall response rate remains around 30–40%, with significant interindividual variability in treatment response [5,6,7]. Combination therapy with other ICIs shows improved efficacy compared to monotherapy, yet a substantial number of patients still fail to respond [8,9]. Furthermore, many HCC patients have cirrhosis due to chronic liver disease [10], and the occurrence of immune-related adverse events (irAEs) can worsen hepatic reserve, sometimes making treatment continuation difficult. These differences in treatment response and the occurrence of adverse events highlight the need to explore new biomarkers that can predict treatment efficacy and irAEs.

Recent studies have demonstrated that the gut microbiota plays a pivotal role in modulating systemic immune responses and shaping the clinical outcomes of cancer therapies, leading to growing interest in the gut microbiota as a potential determinant of ICI efficacy [11,12]. This concept is supported by the gut–liver axis paradigm, in which intestinal microorganisms and their metabolites continuously interact with the liver through the portal circulation, thereby influencing hepatic immunity, immune tolerance, and inflammatory signaling [13]. Dysbiosis of the gut microbiota can disrupt intestinal barrier function and promote the translocation of microbial products, ultimately affecting systemic and hepatic immune responses. In HCC, accumulating evidence suggests that alterations in the composition of the gut microbiota may represent a promising therapeutic target for enhancing the efficacy of immunotherapy. Given that the liver is constantly exposed to microbial-derived antigens and metabolites, gut microbiota-driven modulation of innate and adaptive immune cells, including Kupffer cells, regulatory T cells, and natural killer T cells, may critically influence the tumor immune microenvironment. Indeed, several clinical studies have reported associations between specific gut microbiota signatures and treatment response to ICIs in patients with HCC, as well as correlations with the development of irAEs [14,15,16,17,18,19,20]. However, despite the identification of shared trends across these reports, substantial heterogeneity in patient populations, microbial profiles, and analytical approaches has prevented agreement on a consensus regarding clinically relevant microbial determinants of ICI response in HCC.

Against this background, the present study aimed to clarify the association between treatment response to atezolizumab plus bevacizumab (Atez/Bev) therapy and gut microbiota composition in patients with advanced HCC, within the framework of the gut–liver axis and systemic immune regulation.

## 2. Materials and Methods

### 2.1. Subjects and Ethics

This study was conducted with the approval of the Ethics Committee of Hiroshima University. The subjects included 29 patients who provided stool samples between May 2017 and October 2024 and who received Atez/Bev therapy for advanced HCC. Written informed consent was obtained from all patients. At the time of administration, hepatic reserve function was Child–Pugh class A or B, and performance status was ECOG PS 0 or 1.

### 2.2. Treatment and Clinical Evaluation Methods

Atezolizumab (1200 mg) and bevacizumab (15 mg/kg) were administered intravenously every 3 weeks. Adverse events were evaluated based on the Common Terminology Criteria for Adverse Events (CTCAE ver. 5.0). Dosage was reduced as necessary in accordance with current treatment guidelines in patients who developed drug-related adverse events. Treatment was discontinued in patients who developed serious adverse events. Each treatment was continued until death or until meeting one of the following criteria: disease progression after treatment, occurrence of an adverse event requiring treatment discontinuation, deterioration to an ECOG-PS of 4, or decline in hepatic reserve. HCC diagnosis was based on imaging findings from dynamic CT or MRI scans. Radiographic response was evaluated by dynamic liver CT or MRI 4–6 weeks and 8–12 weeks after treatment initiation and then every 4–8 weeks thereafter. Treatment response was evaluated based on the Response Evaluation Criteria in Solid Tumors (RECIST) version 1.1 and modified RECIST (mRECIST) guidelines, with complete response (CR) or partial response (PR) considered as the responder group and stable disease (SD) or progressive disease (PD) classified as the non-responder group. Overall survival (OS) was defined as the time from treatment initiation to death from any cause. For surviving patients, the censoring date was the last follow-up date. Progression-free survival (PFS) was defined as the time from treatment initiation to the first radiographic progression based on mRECIST or death from any cause. For patients alive without radiographic progression, the censoring date was defined as the date of the last imaging evaluation or the date of switching to another treatment. Liver function was assessed in all patients prior to treatment initiation. Liver reserve capacity was evaluated using the Child–Pugh scoring system and the modified albumin-bilirubin (mALBI) grade. The mALBI grade was developed to more accurately assess patients with conventional ALBI grade 2. The mALBI classification system is based on a 4-tier assessment (ALBI score ≤ −2.60 is Grade 1, > −2.60 to ≤ −2.27 is Grade 2a, > −2.27 to ≤ −1.39 is Grade 2b, > −1.39 is Grade 3).

### 2.3. Collection of Fecal Samples and Gut Microbiota Analysis

Fecal samples were collected using a stool collection tube prior to treatment initiation and stored at −80 °C until DNA extraction. PCR amplification targeted the V1-V2 region of the 16S rRNA gene [21,22], and sequencing was performed using an Ion Personal Genome Machine (Ion PGM™; Thermo Fisher Scientific, Waltham, MA, USA). Bacterial identification was performed using QIIME2 software version 2025.7 (https://qiime2.org/). The relative abundance of genera in each sample was calculated from the operational taxonomic units (OTUs) obtained for each case and used for analysis.

### 2.4. Statistical Analysis

Continuous variables are expressed as median and interquartile range (IQR), and categorical variables as absolute and relative frequencies. The Mann–Whitney *U*-test was used to compare continuous data. The chi-square test was used to analyze categorical data. The area under the curve (AUC) for evaluating the discriminatory ability of each gut bacterium to predict treatment response was analyzed using receiver operating characteristic (ROC) curves, and statistical significance was tested against the null hypothesis (AUC = 0.5) using the Mann–Whitney *U*-test. The cutoff values for discrimination were determined using the Youden index, which was calculated by adding sensitivity and specificity and then subtracting 1 from the sum. Univariate and multivariate logistic regression analyses were used to examine categorical variables associated with the differences between the responder group and the non-responder group. In multivariate analysis, a stepwise selection procedure was used (*p* value threshold: probability to enter and probability to leave were 0.05 and 0.10, respectively). Kaplan–Meier survival curves and the log-rank test were used to estimate PFS. Multivariate analysis was conducted with a Cox proportional hazards model with a stepwise selection procedure based on the same criteria as above. Factors showing *p* < 0.1 in univariate analysis were subsequently included in multivariate analysis. A *p* value < 0.05 was considered statistically significant. Statistical analysis was performed using SPSS 29.0 (IBM Corp., Armonk, NY, USA).

## 3. Results

### 3.1. Patient Background Characteristics

Of the 29 total cases, 15 (51.7%) belonged to the responder group, and 14 (48.3%) were in the non-responder group. The demographic and clinical characteristics of these patients are summarized in Table 1. For all 29 cases, the median age was 73 years, and 23 (79%) were male. mALBI grades of 1, 2a, and 2b were noted in 11 (37.9%), 5 (17.2%), and 13 (44.9%) cases, respectively. Child–Pugh class A and B were noted in 24 (82.7%) and 5 (17.3%) cases, respectively. The ECOG-PS was 0 in twenty-seven patients (93.1%) and 1 in two patients (6.9%). Furthermore, two patients (6.9%) had macroscopic vascular invasion, and ten (34.4%) had extrahepatic metastasis. BCLC stage was 4 (13.8%) in A, 13 (44.8%) in B, and 12 (41.4%) in C. Atez/Bev was administered as first-line therapy in 14 cases (48.3%) and as subsequent-line therapy in 15 cases (51.7%). Proton pump inhibitors (PPIs) or potassium-competitive acid blockers (P-CABs) were administered in 17 cases (58.6%). The median period from sample collection to initiation of Atez/Bev was 50.4 months, the median PFS was 5.87 months, and the median OS was 15.8 months. When patients in the responder group were compared with those in the non-responder group, statistically significant differences were observed in extrahepatic metastasis (*p* = 0.021), BCLC stage (*p* = 0.012), PFS (*p* < 0.001), and OS (*p* = 0.026).

### 3.2. Composition of the Gut Microbiota in Each Case

**Figure 1:**
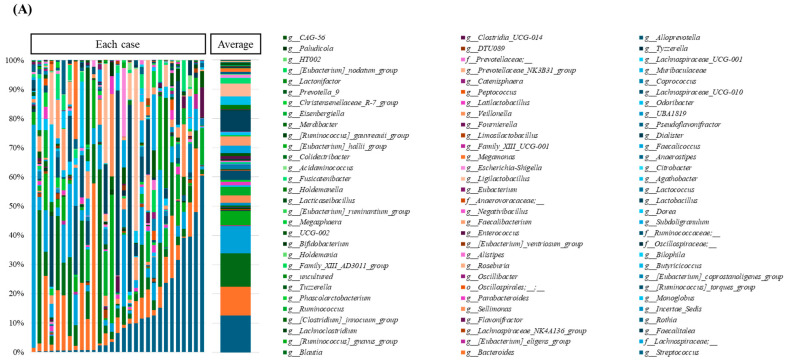

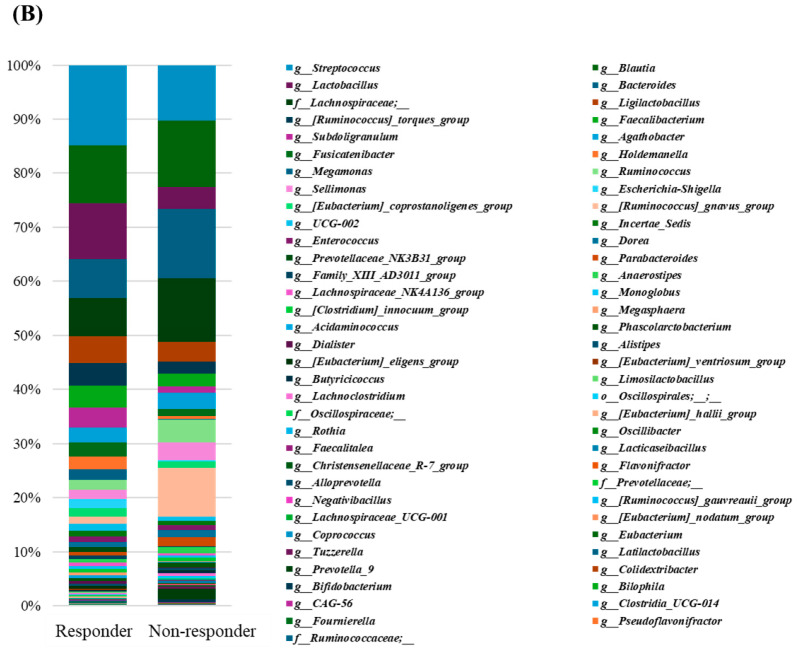
Relative abundance of gut microbiota in 29 patients: (**A**) Percentage of bacteria at the discernible genus level in the total stool of each patient and the average. The names of the bacterial genera shown in the bar chart are listed next to the chart. (**B**) Percentage of bacterial genera exhibiting a prevalence of 0.1% or higher in the responder (*n* = 15) and non-responder groups (*n* = 14). The names of the bacterial genera are listed on the righthand side of the chart.

Phylogenetic analysis identified 5 phyla, 7 classes, 17 orders, 29 families, and 90 genera of gut bacteria. Figure 1A show the genus-level bacterial community composition in stool samples from HCC patients (*n* = 29) before starting Atez/Bev administration. As illustrated in the bar chart, there are a wide variety of bacterial genera, and there are considerable individual differences in their prevalence, indicating that the composition of bacterial genera varies substantially between individuals. While it is difficult to fully capture this individual diversity, we divided the patients into two groups and calculated the average values in order to compare the treatment response. Figure 1B shows the average values of bacterial genera exhibiting a prevalence of 0.1% or higher in the responder (*n* = 15) and non-responder groups (*n* = 14). The average value of each bacterium is shown as a bar chart, with the vertical axis representing the relative abundance of bacteria distinguishable at the genus level, totaling 100% for all bacteria. Individual bacteria are color-coded. The top five genera in the responder group were *Streptococcus* (14.8%), *Blautia* (10.8%), *Lactobacillus* (10.3%), *Bacteroides* (7.2%), and an unclassified genus within the *Lachnospiraceae* family (7.1%). In the non-responder group, the top five were *Bacteroides* (12.8%), *Blautia* (12.2%), *Lachnospiraceae* family unclassified genus (11.7%), *Streptococcus* (10.3%), and *[Ruminococcus]_gnavus_group* (9.0%). The responder group had a higher proportion of *Streptococcus* (14.8% vs. 10.3%) and *Lactobacillus* (10.3% vs. 4.1%) compared to the non-responder group. Conversely, the non-responder group showed higher proportions of *Bacteroides* (12.8% vs. 7.2%) and an unclassified genus within the *Lachnospiraceae* family (10.3% vs. 4.1%) relative to the responder group.

### 3.3. Investigation of Gut Bacteria Contributing to Response and PFS Using ROC Curves

**Figure 2:**
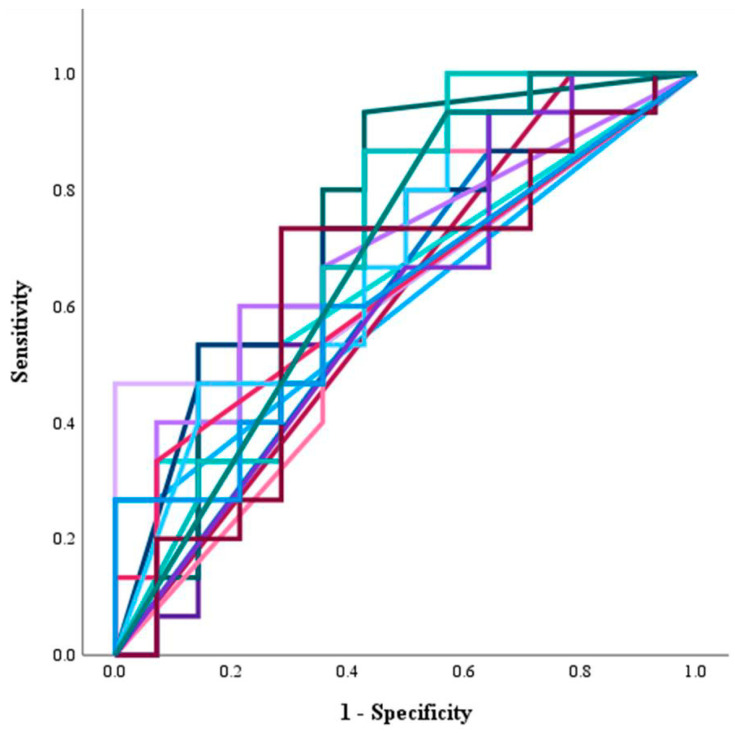
Evaluation of the ability to distinguish between the responder and non-responder groups based on ROC curves constructed for all bacterial genera obtained from 29 patients. The results are shown for 18 genera with an AUC > 0.6. The results for all 90 bacterial genera are shown in Figure A1 in Appendix A.

**Figure 3:**
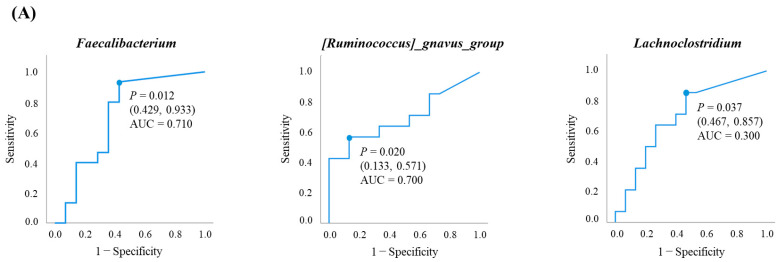

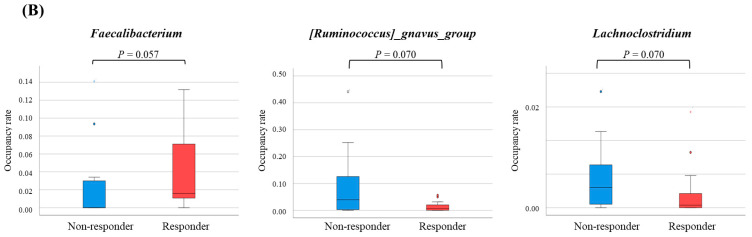
Bacteria capable of distinguishing between the responder and non-responder groups: (**A**) The AUC of the ROC curves for *Faecalibacterium*; *Ruminococcus gnavus group*; and *Lachnoclostridium* were 0.71; 0.30; and 0.30; respectively. *p* values were calculated using Mann–Whitney *U*-tests. (**B**) Box-and-whisker plots showing the occupancy rates of three bacterial genera in the non-responder and responder groups. The non-responder group is represented in blue and the responder group in red. *p* values were calculated using Mann–Whitney *U*-tests.

**Figure A1:**
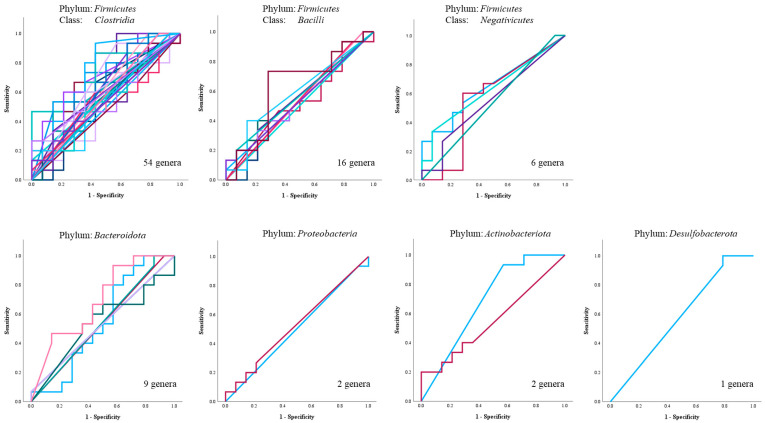
Evaluation of the ability to distinguish between the responder and non-responder groups based on ROC curves constructed for all 90 bacterial genera obtained from 29 patients. Because of the large number of curves, we have classified the 90 genera by phylum to make the data easier to read. However, since the *Firmicutes* phylum contains more bacterial species than the other phyla, we have presented it at the lower taxonomic level of class rather than phylum (the three figures in the top row). As some curves overlap completely, we have also indicated the number of genera included in each figure within the figure itself.

Using the prevalence rate of 90 bacterial genera, we analyzed the discriminatory ability between responders and non-responders using ROC curves. We identified 18 bacterial genera with an AUC of 0.6 or higher (Table 2). In Figure 2, ROC curves are shown for 18 genera with an AUC > 0.6. The results for all 90 bacterial genera are shown in Figure A1 in Appendix A. Using the prevalence rate that yielded the maximum Youden index as the cutoff value, we analyzed the discriminatory ability between the responder and non-responder groups, as well as the correlation with prolonged PFS. Statistically significant differences were observed for higher *Faecalibacterium* (*p* = 0.012), lower *[Ruminococcus]_gnavus_group* (*p* = 0.020), and lower *Lachnoclostridium* (*p* = 0.037) in distinguishing responders from non-responders. The respective ROC curves are shown in Figure 3A. Interestingly, these three bacterial genera were all classified within the same phylum and the same class, namely the *Clostridia* class of the *Firmicutes* phylum (Figure A1). Although not statistically significant, a slightly higher prevalence of *Faecalibacterium* was observed in the responder group, while a higher prevalence of *[Ruminococcus]_gnavus_group* and *Lachnoclostridium* was observed in the non-responder group (Figure 3B). Next, multivariate analysis incorporating clinical prognostic factors was performed to assess the discriminatory ability of the three bacterial genera (high vs. low) for distinguishing responders from non-responders. Continuous variables were converted to binary variables using the median values from the 29 cases. Multivariate analysis included variables with *p* < 0.1 in univariate analysis: presence/absence of extrahepatic metastasis, BCLC stage (A or B vs. C), first-line vs. later-line treatment, *Faecalibacterium*-high vs. -low, *[Ruminococcus]_gnavus_group*-high vs. -low, *Lachnoclostridium*-high vs. -low, mALBI grade (1 or 2a vs. 2b). Binary logistic regression analysis using the likelihood ratio test for variable reduction identified BCLC stage C (*p* = 0.036) and *Faecalibacterium*-high (*p* = 0.016) as statistically significant independent contributing factors (Table 3).

**Figure 4:**
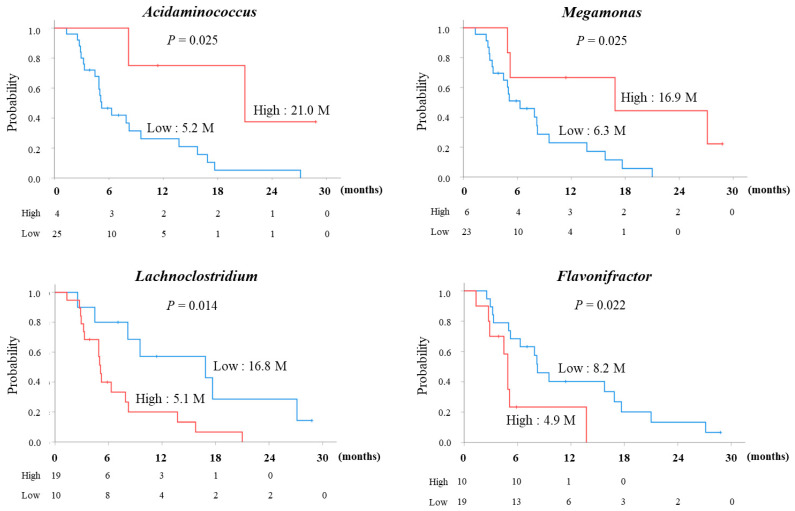
Comparison of PFS survival curves between high and low groups for four bacterial genera. The cutoff values were determined using the AUC of the ROC curve that maximized the Youden index. On the survival curve, the group with the higher prevalence is indicated in red, while the blue line indicates the group with lower prevalence. The median PFS for each group is noted (months). *p* values were calculated using the log-rank tests.

Next, Kaplan–Meier curves for PFS were constructed for the high and low groups of each bacterium, using the relative abundance of the 18 bacterial genera in Table 2, with cutoff values determined based on the maximum Youden index. As a result, significant differences were observed in four bacterial genera (Figure 4). The *Acidaminococcus*-high group (*p* = 0.025) and the *Megamonas*-high group (*p* = 0.025) had significantly better PFS compared to their respective low groups. Furthermore, the *Lachnoclostridium*-low group (*p* = 0.014) and the *Flavonifractor*-low group (*p* = 0.022) also had significantly better PFS compared to their respective high groups. Multivariate analysis was performed using these factors. Univariate analysis identified the following factors with *p* < 0.1: intrahepatic tumor size (≤30 mm vs. >30 mm), BCLC stage (A or B vs. C), first-line vs. later-line treatment, *Lachnoclostridium*-high vs. -low, *Flavonifractor*-high vs. -low, *Acidaminococcus*-high vs. -low, and *Megamonas*-high vs. -low. In Table 4, Cox regression analysis using the likelihood ratio with variable reduction showed statistically significant correlations for BCLC stage C (*p* = 0.003), *Acidamonococcus*-high (*p* = 0.013), and *Megamonas*-high (*p* = 0.029).

## 4. Discussion

This study demonstrated that a high relative abundance of *Faecalibacterium* in the gut microbiota correlates with response to Atez/Bev treatment in HCC. In a different cancer type, Gopalakrishnan et al. reported in their analysis of the gut microbiota of 112 patients with malignant melanoma that *Faecalibacterium* was relatively more abundant in the responder group than in the non-responder group [23]. Furthermore, patients whose *Faecalibacterium* relative abundance exceeded the median had longer PFS compared to those whose abundance did not exceed the median. In addition, they demonstrated in mouse experiments that mice transplanted with fecal matter from responders exhibited significantly reduced tumor growth compared to those transplanted with non-responder fecal matter and showed significantly higher abundance of *Faecalibacterium* in the gut microbiota. They concluded that patients with gut microbiota exhibiting a higher proportion of *Ruminococcaceae* and *Faecalibacterium* may have enhanced systemic and antitumor immune responses mediated by increased antigen presentation, along with improved effector T cell function in peripheral and tumor microenvironments. Supporting the relevance of these findings, multiple studies have reported a statistically significant positive correlation between high proportions of *Faecalibacterium* and the *Ruminococcaceae* family (which includes *Faecalibacterium*) and CD8+ T cell tumor infiltration, suggesting an association [24,25].

In this study, patients with a higher abundance of *Acidaminococcus* and *Megamonus* showed significantly prolonged PFS, suggesting that gut microbiota composition may influence the treatment response to ICIs. Regarding HCC, a report on 36 HCC patients undergoing ICI therapy showed that *Acidaminococcus* was detected at a higher rate in the disease-control group (CR/PR or long-term SD) than in the non-disease-control group, suggesting a potential positive association between this genus and ICI treatment response [16]. Furthermore, Hamada et al. analyzed 26 ICI-treated patients with primarily lung and kidney cancers and reported that a high prevalence of *Acidaminococcus* was associated with both increased irAEs and treatment response [20]. The presence of *Acidaminococcus* is thought to characterize a “high-response, high-toxicity” type of immune activation profile. This report is consistent with the association with prolonged PFS observed in this study and supports the possibility that *Acidaminococcus* functions as an intestinal bacterium that determines the degree of immune activation.

Furthermore, *Acidaminococcus*, *Megamonus*, and *Faecalibacterium* are implicated in the production of short-chain fatty acids (SCFAs), and *Acidaminococcus* and *Megamonus* have been suggested to contribute to the production of acetate and propionate, which are types of SCFAs [26]. High concentrations of SCFAs in feces or plasma are associated with response to PD-1 inhibitor therapy and prolonged progression-free survival [27]. Butyrate has been shown to inhibit histone deacetylases [28,29,30], increase PD-1 ligand expression in melanoma cells, enhance the response to immunotherapy, suppress apoptosis of intratumoral CD4+ T cells, elevate antitumor immune responses, and inhibit tumor proliferation [31,32,33].

However, there is a study that contradicts our result, reporting a relatively high relative abundance of *Megamonus* in the non-responder group of advanced HCC patients treated with nivolumab [18]. Possible reasons for these differences include regional variations in the gut microbiota and changes in the gut microbiota due to liver disease progression. It is crucial to determine whether reproducibility can be achieved with future data.

On the other hand, the *Lachnoclostridium* and the *Ruminococcus gnavus group* showed a tendency to be elevated in the non-responder group. The *Lachnoclostridium*-low group demonstrated a significant prolongation in PFS. Few reports have shown the distribution of these bacterial genera in HCC patients undergoing Atez/Bev therapy. Lee et al. analyzed 94 patients with HCC who received ICIs (nivolumab or pembrolizumab) [17]. They demonstrated that the *Lachnoclostridium* genus and *Ruminococcus gnavus group* were predominant in responders, with fecal abundance showing a strong positive correlation with secondary bile acids, such as ursodeoxycholic acid (UDCA), tauro-UDCA, ursocholic acid, and murideoxycholic acid. The authors suggest that *Lachnoclostridium* may influence the hepatic immune environment via the bile acid metabolic pathway, potentially enhancing the efficacy of ICI treatment. Notably, *Lachnoclostridium* shares high genetic homology with the *Ruminococcus gnavus group*, suggesting that both may play a common role in bile acid metabolism and immune response regulation. However, in our study, both *Lachnoclostridium* and the *Ruminococcus gnavus group* were over-represented in the non-responder group, suggesting that these genera might instead be associated with ICI treatment resistance and poor prognosis. For the *Ruminococcus gnavus group*, reports indicate a higher proportion in non-responders to ICI therapy (nivolumab) in 8 advanced HCC cases [28] and a correlation with shorter PFS in 27 malignant melanoma cases treated with ICI (anti-PD1, anti-CTLA4, and anti-PD1/CTLA4) [34]. Additionally, studies using mouse models have reported that the *Ruminococcus gnavus group* shifts intestinal immunity toward “immune tolerance (suppression)” via bile acids [35]. However, the community composition of multiple taxa may be more important than the prevalence of any single species, making it difficult to provide sufficient evidence for ICI treatment resistance.

If we can infer immune states in which ICI efficacy is likely to be demonstrated or where irAEs are likely to occur through gut microbiota analysis, it may be possible to induce favorable conditions using diverse therapies, including gut microbiota modification. At the very least, utilizing these bacteria as biomarkers could enable the development of case-specific treatment strategies, such as selecting drug therapies for HCC.

Finally, it must be noted that this study has many limitations, and it is reasonable to consider it as one result of exploratory research at this stage. Among the limitations are the failure to fully account for the various factors that influence the gut microbiota, and the long median interval of 50.4 months between stool sample collection and the start of treatment. Generally, it has been reported that the gut microbiota in adults, while subject to changes due to multiple factors such as antibiotics, diet, and disease, remains relatively stable due to its resilience [36,37,38]. That said, it is well known that diet [39,40], the progression of liver disease [13,41,42], aging [43,44], and medication administration [45,46,47,48,49] influence the composition of the gut microbiota, and these factors may have influenced the results of this study as confounding variables. The subjects in this study were patients who continued to receive care at our department from the time of stool sample collection through the initiation of Atez/Bev treatment, and according to medical interviews, there were no significant changes in their dietary habits. No concomitant gastrointestinal diseases were observed during the observation period. Regarding drug administration, we confirmed that no antibiotics, including rifaximin, were administered during the observation period. No significant differences were observed in the administration of PPIs or P-CABs in this study (Table 1 and Table 3). However, even these findings must be interpreted within the context of the most significant limitation of this study. Namely, this study is a small-sample, single-center, retrospective study. In addition to the lack of statistical power due to the small sample size, the large number of bacterial genera examined under multiple testing could have resulted in false-positive findings. Regarding the AUC threshold, we arbitrarily set it to 0.6 to identify potential candidates, taking into account that the influence of individual bacterial genera is relatively weak; therefore, we cannot rule out the possibility of an increased rate of false positives. Furthermore, in multivariate analyses that include clinical factors, inclusion of a large number of variables has the potential to destabilize the regression model. In order to ensure that important confounding and independent factors were not overlooked, we included variables with *p* < 0.1 (rather than *p* < 0.05) from the univariate analysis into the multivariate analysis, and then further narrowed down the variables using stepwise selection. However, we do not believe that this alone was sufficient to adequately adjust for background factors. For example, it seems unnatural that factors such as intrahepatic tumor size and whether patients received first-line therapy or subsequent treatments did not remain as independent predictors in the multivariate analysis. We suspect that these factors were confounded with the BCLC stage, which remained as an independent predictor. In any case, the sample size was insufficient to perform propensity score matching between the two groups, and this study was not designed as a prospective study with a sufficient sample size to standardize important patient background factors in advance. Therefore, it is appropriate to regard these results as preliminary, as they leave open the possibility of false positives or overfitting. As described above, given the many limitations of this study, the suggestive results obtained require further verification through future analyses involving a larger number of cases and confirmation of reproducibility in independent sample sets. Finally, there is one more point regarding the experimental methodology that cannot be overlooked: this study did not include negative controls in the 16S rRNA sequencing analysis. Given the small sample size and the risk of reagent contamination in such microbiome studies, it cannot be ruled out that these factors may have influenced the results.

Our exploratory analysis has identified several bacterial genera that may be associated with the response to Atez/Bev therapy. Although some uncertainty remains due to limitations in the sample size and study design, we hope that these findings will contribute to our understanding of the gut–liver axis paradigm and help elucidate the multifaceted roles of the gut microbiota in influencing systemic immune responses and the response to HCC treatment.

## 5. Conclusions

In this study, we observed that the presence of a high relative abundance of *Faecalibacterium* in patients with advanced HCC prior to Atez/Bev administration was associated with a favorable response, whereas a high relative abundance of *Acidaminococcus* and *Megamonus* was associated with prolonged PFS. Conversely, the presence of *Lachnoclostridium* and *[Ruminococcus]_gnavus_group* was observed to be associated with limited response to Atez/Bev.

## Figures and Tables

**Table 1:** Clinical characteristics at initiation of Atez/Bev (*n* = 29).

		Responder (CR/PR)	Non-Responder (SD/PD)	
	*n* = 29	*n* = 15	*n* = 14	*p*
Age (IQR); years	73 (66.5–81)	75 (69–82)	72 (61.75–80.5)	0.591 *
Gender (male/female); *n*	23/6	11/4	12/2	0.651 **
Etiology (HBV/HCV/NBNC); *n*	3/16/10	2/8/5	1/8/5	0.780 *
Modified ALBI grade (1/2a/2b); *n*	11/5/13	6/2/7	5/3/6	1.000 *
Child–Pugh class (A/B); *n*	24/5	12/3	12/2	1.000 **
Performance status (0/1); *n*	27/2	15/0	12/2	0.224 **
Macroscopic vascular invasion (absent/present); *n*	27/2	14/1	12/2	0.598 **
Extrahepatic metastasis (absent/present); *n*	19/10	13/2	6/8	0.021 **
Size of intrahepatic tumor (IQR); mm	30 (16.5–39)	30 (17–35)	31 (15–44.75)	0.780 *
BCLC stage (A/B/C); *n*	4/13/12	4/8/3	0/5/9	0.012 *
Serum AFP (IQR); ng/mL	33.8 (3.6–134)	25 (3.6–76.8)	36.2 (2.48–683.58)	0.949 *
Serum AFP-L3 (IQR); %	14.6 (0.5–35.75)	9.15 (0.5–27.75)	22.55 (0.5–52.05)	0.667 *
Serum DCP (IQR); mAU/mL	343.0 (36–6693.5)	133 (50–1832)	1873.5 (31.75–8984.5)	0.270 *
1st line/later line; *n*	14/15	9/6	4/10	0.089 **
PPI or P-CAB administration (with/without); *n*	17/12	10/5	7/7	0.462 **
Period from sample collection to initiation of Atez/Bev; months	50.4 (38.28–60.57)	47.6667 (35.7–59.53)	52.15 (39.78–70.32)	0.591 *
PFS; months	5.87 (3.58–12.57)	9.53 (5.87–17.7)	4.17 (2.83–5.88)	<0.001 *
OS; months	15.8 (8.97–26.03)	23.0 (15.8–32.13)	10.73 (7.18–17.23)	0.026 *

Continuous variables are presented as median and interquartile range (IQR). CR: complete response; PR: partial response; SD: stable disease; PD: progressive disease; Atez/Bev: atezolizumab/bevacizumab; HBV: hepatitis B viral infection; HCV: hepatitis C viral infection; NBNC: non-B non-C; BCLC: Barcelona Clinic Liver Cancer; AFP: alpha-fetoprotein; DCP: des-γ-carboxyprothrombin; PPI: proton pump inhibitor; P-CAB: potassium-competitive acid blocker; PFS: progression-free survival; OS: overall survival. *p* values were calculated by * Mann–Whitney *U*-tests and ** chi-square tests.

**Table 2:** Bacterial genera with AUC exceeding 0, along with their respective AUC values.

Bacterial Genera	AUC
*g__Faecalibacterium*	0.710
*g__[Ruminococcus]_gnavus_group*	0.700
*g__Lachnoclostridium*	0.700
*g__Parabacteroides*	0.690
*g__Tuzzerella*	0.686
*g__Bifidobacterium*	0.686
*g__Lachnospiraceae_NK4A136_group*	0.679
*g__[Eubacterium]_ventriosum_group*	0.660
*g__Acidaminococcus*	0.648
*g__Flavonifractor*	0.643
*g__Colidextribacter*	0.631
*g__Streptococcus*	0.629
*g__Megamonas*	0.629
*g__Subdoligranulum*	0.619
*g__Ruminococcus*	0.610
*f__Anaerovoracaceae;__*	0.607
*g__Agathobacter*	0.607
*f__Ruminococcaceae;__*	0.607

AUC: area under the curve.

**Table 3:** Analysis of factors related to response and non-response to Atez/Bev therapy (*n* = 29).

Variable	Univariate Analysis			Multivariate Analysis		
	OR	95%CI	*p* *	OR	95%CI	*p* **
PS (0 vs. 1)	-	-	0.999			
Age (≤73 vs. >73 years)	1.524	0.352–6.601	0.573			
Gender (male vs. female)	0.458	0.070–3.017	0.417			
Etiology (viral hepatitis vs. NBNC)	1.111	0.240–5.142	0.893			
Modified ALBI grade (1 or 2a vs. 2b)	1.167	0.269–5.054	0.837			
Child–Pugh class (A vs. B)	1.5	0.211–10.649	0.685			
Macroscopic vascular invasion (absent vs. present)	0.429	0.034–5.333	0.51			
Extrahepatic metastasis (absent vs. present)	0.115	0.019–0.717	0.02			
Size of intrahepatic tumor (≤30 vs. >30 mm)	2.019 × 10^9^	0.000–∞	0.999			
BCLC stage (A or B vs. C)	0.139	0.026–0.739	0.021	0.114	0.015–0.863	0.036
Serum AFP (≤33.8 vs. >33.8 ng/mL)	0.500	0.114–2.194	0.358			
Serum AFP-L3 (≤14.6 vs. >14.6%)	0.656	0.151–2.843	0.573			
Serum DCP (≤343 vs. >343 mAU/mL)	0.278	0.060–1.286	0.101			
1st line vs. later line	0.267	0.056–1.260	0.095			
PPI or P-CAB administration (with vs. without)	2.00	0.446–8.963	0.365			
Period from stool sample collection to initiation of Atez/Bev (≤50.4 vs. >50.4 months)	0.875	0.204–3.761	0.858			
*Faecalibacterium* (high vs. low)	18.667	1.894–184.017	0.012	22.306	1.793–277.499	0.016
*[Ruminococcus]_gnavus_group* (high vs. low)	0.115	0.019–0.717	0.02			
*Lachnoclostridium* (high vs. low)	0.146	0.024–0.890	0.037			

Continuous variables are presented as the median and interquartile range (IQR). OR: odds ratio; CI: confidence interval; PS: performance status; Atez/Bev: atezolizumab/bevacizumab; NBNC: non-B non-C; BCLC: Barcelona Clinic Liver Cancer; AFP: alpha-fetoprotein; DCP: des-γ-carboxy prothrombin; PPI: proton pump inhibitor; P-CAB: potassium-competitive acid blocker. *p* values were calculated by * univariate and ** multivariate logistic regression analyses. Regarding PS and the size of the intrahepatic tumor, OR and/or 95%CI could not be estimated due to sparse data.

**Table 4:** Analysis of factors associated with PFS, including the high vs. low prevalence of selected bacterial genera (*n* = 29).

	Univariate	Multivariate		
	*p* *	HR	95%CI	*p* **
PS (0 vs. 1)	0.460			
Age (≤73 vs. >73 years)	0.909			
Gender (male vs. female)	0.970			
Etiology (viral hepatitis vs. NBNC)	0.285			
modified ALBI grade (1 or 2a vs. 2b)	0.697			
Child–Pugh class (A vs. B)	0.591			
Macroscopic vascular invasion (absent vs. present)	0.317			
Extrahepatic metastasis (absent vs. present)	0.122			
Size of intrahepatic tumor (≤30 vs. >30 mm)	0.001			
BCLC stage (A or B vs. C)	0.089	4.342	1.659–11.362	0.003
Serum AFP (≤33.8 vs. >33.8 ng/mL)	0.923			
Serum AFP-L3 (≤14.6 vs. >14.6%)	0.687			
Serum DCP (≤343 vs. >343 mAU/mL)	0.245			
1st line vs. later line	0.009			
*Lachnoclostridium* (high vs. low)	0.014			
*Flavonifractor* (high vs. low)	0.022			
*Acidaminococcus* (high vs. low)	0.025	0.122	0.023–0.639	0.013
*Megamonas* (high vs. low)	0.025	0.222	0.057–0.860	0.029

Continuous variables are presented as the median and interquartile range (IQR). HR: hazard ratio; CI: confidence interval; PS: performance status; NBNC: non-B-non-C; BCLC: Barcelona Clinic Liver Cancer; AFP: alpha-fetoprotein; DCP: des-γ-carboxy prothrombin. *p* values were calculated by * log-rank tests and ** Cox regression analyses.

## Data Availability

The data presented in this study are available on request from the corresponding author due to ethical restrictions.

## Abbreviations

The following abbreviations are used in this manuscript:

Atez/Bev	atezolizumab plus bevacizumab combination therapy
HCC	hepatocellular carcinoma
PFS	progression-free survival
ICI	immune checkpoint inhibitor
irAE	immune-related adverse event
RECIST	Response Evaluation Criteria in Solid Tumors
mRECIST	modified RECIST
CR	complete response
PR	partial response
SD	stable disease
PD	progressive disease
OS	overall survival
mALBI	modified albumin-bilirubin
OTU	operational taxonomic unit
IQR	interquartile range
AUC	area under the curve
ROC	receiver operating characteristic
PPI	proton pump inhibitor
P-CAB	potassium-competitive acid blocker
SCFA	short-chain fatty acid
UDCA	ursodeoxycholic acid
